# Exhaled volatile organic compounds for diagnosis of hepatocellular carcinoma

**DOI:** 10.1038/s41598-022-08678-z

**Published:** 2022-03-29

**Authors:** Thanikan Sukaram, Rossarin Tansawat, Terapap Apiparakoon, Thodsawit Tiyarattanachai, Sanparith Marukatat, Rungsun Rerknimitr, Roongruedee Chaiteerakij

**Affiliations:** 1 Division of Gastroenterology, Department of Medicine, Faculty of Medicine, Chulalongkorn University, and King Chulalongkorn Memorial Hospital, Thai Red Cross Society, Bangkok, Thailand; 2 Department of Food and Pharmaceutical Chemistry, Faculty of Pharmaceutical Sciences, Chulalongkorn University, Bangkok, Thailand; 3 Center of Excellence for Innovation and Endoscopy in Gastrointestinal Oncology, Division of Gastroenterology, Faculty of Medicine, Chulalongkorn University, Bangkok, Thailand; 4 Department of Computer Engineering, Faculty of Engineering, Chulalongkorn University, Bangkok, Thailand; 5 Faculty of Medicine, Chulalongkorn University, Bangkok, Thailand; 6 Image Processing and Understanding Team, Artificial Intelligence Research Group, National Electronics and Computer Technology Center (NECTEC), Bangkok, Thailand

**Keywords:** Metabolomics, Cancer metabolism, Cancer screening, Tumour biomarkers, Biomarkers

## Abstract

Volatile organic compounds (VOCs) profile for diagnosis and monitoring therapeutic response of hepatocellular carcinoma (HCC) has not been well studied. We determined VOCs profile in exhaled breath of 97 HCC patients and 111 controls using gas chromatography–mass spectrometry and Support Vector Machine algorithm. The combination of acetone, 1,4-pentadiene, methylene chloride, benzene, phenol and allyl methyl sulfide provided the highest accuracy of 79.6%, with 76.5% sensitivity and 82.7% specificity in the training set; and 55.4% accuracy, 44.0% sensitivity, and 75.0% specificity in the test set. This combination was correlated with the HCC stages demonstrating by the increased distance from the classification boundary when the stage advanced. For early HCC detection, d-limonene provided a 62.8% sensitivity, 51.8% specificity and 54.9% accuracy. The levels of acetone, butane and dimethyl sulfide were significantly altered after treatment. Patients with complete response had a greater decreased acetone level than those with remaining tumor post-treatment (73.38 ± 56.76 vs. 17.11 ± 58.86 (× 10^6^ AU, *p* = 0.006). Using a cutoff of 35.9 × 10^6^ AU, the reduction in acetone level predicted treatment response with 77.3% sensitivity, 83.3% specificity, 79.4%, accuracy, and AUC of 0.784. This study demonstrates the feasibility of exhaled VOCs as a non-invasive tool for diagnosis, monitoring of HCC progression and treatment response.

## Introduction

Hepatocellular carcinoma (HCC) is the second major cause of cancer death worldwide^1,2^. It commonly occurs in individuals with cirrhosis and chronic liver diseases, particularly chronic viral hepatitis B and C (HBV and HCV) infection, alcoholic liver disease, and non-alcoholic steatohepatitis (NASH)^3^. Screening and surveillance for HCC is recommended in these at-risk individuals. Upper abdominal ultrasonography is the most commonly used surveillance tool, which has shown high specificity of 92%^4^. However, its performance is operator-dependent with limited sensitivity of 47% for detection of early-stage HCC^4,5^. Serum tumor marker alpha-fetoprotein (AFP) is another tool widely used for HCC detection. Serum AFP at the cutoff value of ≥ 20 ng/mL was shown to yield a sensitivity and specificity of 52% and 94%, and 44% and 85%, for detecting any stage HCC and early stage HCC, respectively^6^. Serum AFP used in combination with ultrasonography slightly improves the detection rate of early HCC, but performance remains low, with a sensitivity of 63%^4^. Radiologic imaging CT or MRI plays a critical role for assessing response to HCC therapy^7^. However, these techniques are expensive and have some adverse effects. New methods for early detection and monitoring of therapeutic response of HCC are therefore needed.

The analysis of volatile organic compounds (VOCs) has gained attention as a novel method for diagnosis of several diseases^8^. The VOCs profile mirrors biological processes typical of different pathologies because VOCs link directly to intracellular metabolic activities including cell death, oxidative stress, or inflammation. VOCs are released from cells into blood circulation and excreted through body fluids, including bile, urine, feces and breath^9^. A number of VOCs were shown to be commonly present in several cancers including colon, lung, pancreas, breast and cholangiocarcinoma^10–14^. The role of VOCs as a diagnostic or screening tool for these cancers has been extensively studied with promising results, but its possible role as a tool for monitoring treatment response has yet been explored.

A number of VOCs were shown to be differentially expressed in HCC. An in vitro study found that HCC cell lines had higher levels of methane-sulfonyl chloride and acetic acid but lower levels of 2,3-di-hydro-benzofuran and ethanol than normal hepatocytes^15^. Another study examining VOCs in HCC patients found that the level of 3-hydroxy-2-butanone was significantly higher in exhaled breath of HCC patients than healthy controls^16^. In a more recent study, the combination of the 3 exhaled VOCs including acetone, acetaldehyde and dimethyl sulfide differentiated HCC from cirrhosis with 72% accuracy, 73% sensitivity and 71% specificity^17^. Although these findings suggested a potential role of VOCs as biomarkers for HCC diagnosis, the number of studies remains sparse. Additionally, it is currently unknown whether the levels of VOCs are related with HCC stages and whether the levels of VOCs change after therapy.

Our study had three goals: 1) To identify the VOC profiles with potential as biomarkers for HCC screening and diagnosis, 2) To determine the correlation between VOC levels and HCC stages, and 3) To measure changes in VOC levels after HCC treatment to explore the feasibility of using VOCs for monitoring treatment response. VOCs in exhaled breath of HCC patients and controls were identified using Gas chromatography-Mass spectrometry (GC–MS). Combinations of VOCs differentiating HCC patients from controls and the correlation between VOC profiles and HCC stages were determined using the Support Vector Machine (SVM) algorithm. Levels of VOCs before and after HCC treatment were evaluated and VOCs levels of HCC patients responding to treatment were compared to those of patients not responding the treatment.

## Methods

The method was performed in accordance with the relevant guidelines and regulations. The study was approved by Institutional Review Board of the Faculty of Medicine, Chulalongkorn University (IRB number. 701/62). The study was conducted in compliance with the International guidelines for human research protection as Declaration of Helsinki, The Belmont Report, CIOMS Guideline and International Conference on Harmonization in Good Clinical Practice (ICH-GCP). All participants gave written informed consent prior to study enrollment.

### Participants

We calculated sample size based on 80% sensitivity, 8% acceptable error and alpha 0.05^18^. Therefore, breath samples were collected from 97 HCC patients and 111 controls (33 healthy volunteers and 78 cirrhosis). The participants were recruited through the Chula Excellence Center of Endoscopy, Division of Gastroenterology, Department of Medicine, Chulalongkorn University. Inclusion criteria for HCC cases were patients newly diagnosed with HCC prior to receiving any treatments. Those who had recurrent HCC or with history of other cancers were excluded. The diagnosis of HCC was made using the American Association for the Study of Liver Diseases criteria: histopathology or typical radiologic images in patients with cirrhosis or chronic HBV infection^19^. The control group included healthy individuals or cirrhotic patients who did not have a history of cancer. Cirrhosis was diagnosed by histopathology or radiologic evidence, including nodular surface of liver, small right liver lobe, caudate lobe or left lobe hypertrophy, in combination with evidence of portal hypertension (varices, collateral vessels, splenomegaly and thrombocytopenia).

**Figure 1:**
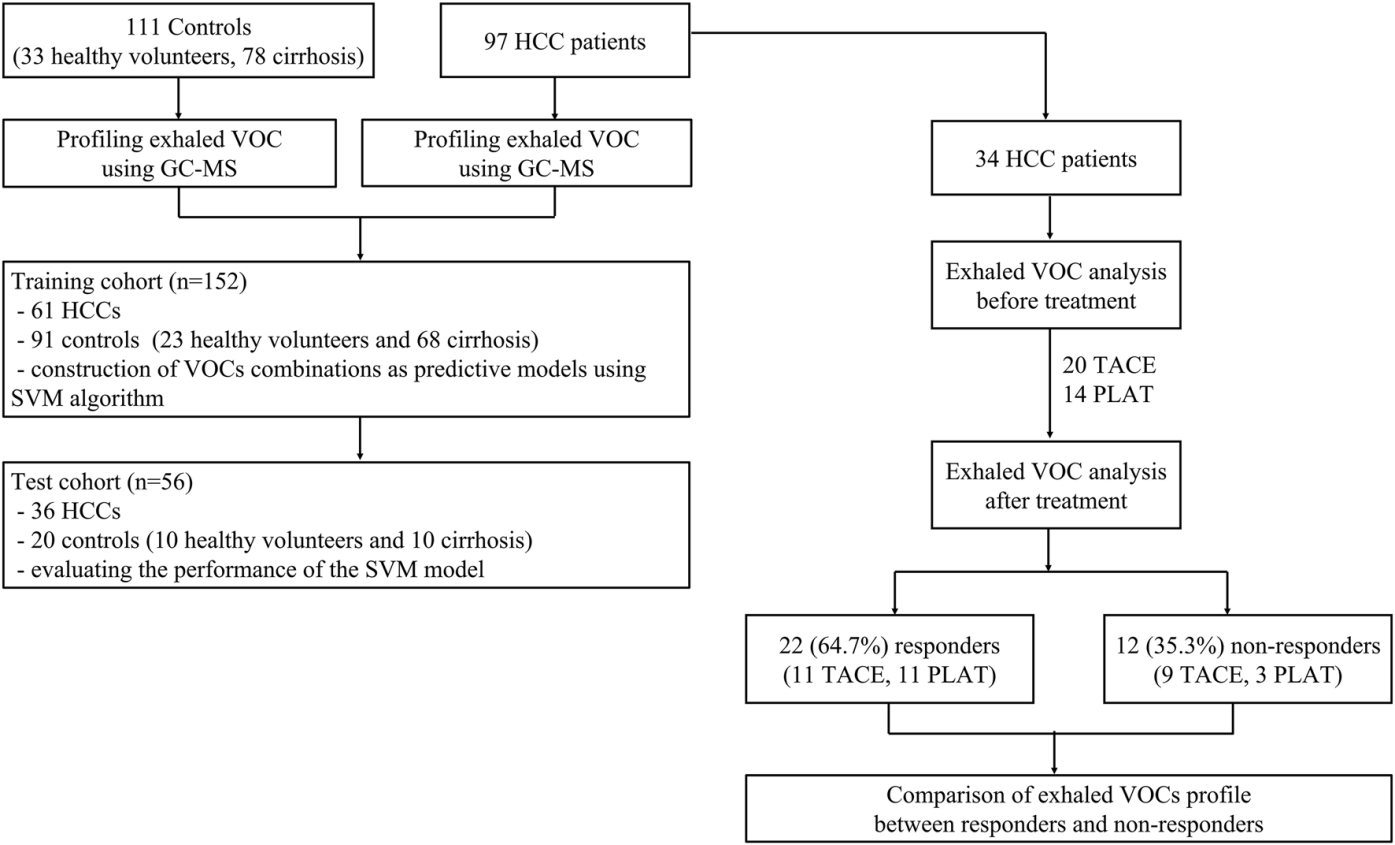
Workflow of the study. GC–MS, Gas chromatography-Mass spectrometry; HCC, Hepatocellular carcinoma; PLAT, Percutaneous Local Ablative Therapy; SVM, Support Vector Machine; TACE, Transarterial Chemoembolization; VOC, Volatile organic compound.

Of the 97 HCC patients, 34 were collected breath samples for follow-up on the changes of VOCs after HCC treatment. The breath samples were collected at 1 day before the patients received treatment and at the time of the imaging study for clinical follow-up visit at 1–2 months post-treatment. Workflow of the patient enrollment process is illustrated in Fig. 1.

### Data collection

We abstracted patient demographics, clinical information including age, gender, smoking status, alcohol intake, underlying diseases (cirrhosis, chronic HBV/HCV infection, and diabetes), and current medications, and laboratory data including liver chemistries and AFP level from electronic medical records. The stages of HCC were classified according to the Barcelona-Clinic Liver Cancer (BCLC) staging system. The BCLC staging system considers 3 main factors including tumor burden, liver function and patient performance status, and classifies HCC into 5 stages: stage 0 (very early), A (early), B (intermediate), C (advanced) and D (terminal)^20^. Tumor response after therapy was evaluated by imaging technique including CT and MRI.

### Breath collection

We applied the protocol for breath collection previously published with some modifications^21,22^. All patients were ceased smoking and alcohol drinking at least 1 day and fasted for a minimum of 8 h before breath sampling to minimize contamination from oral cavity or the effects of exogenous confounders from dietary intakes, smoking and alcohol. The use of antibiotics and probiotics was avoided for 3 weeks prior to the breath sample collection. After fasting for at least 8 h, participants stayed in a 25 °C dedicated room for at least 10 min before breath collection. All participants exhaled their breath with the full expiratory vital capacity into a 1-L disposable Tedlar Bag via disposable mouthpiece (SKC, Inc., USA.) in a single exhalation. The bag was immediately transferred on ice to the Pharmaceutical Research Instrument Center, Faculty of Pharmaceutical Science, Chulalongkorn University. Breath samples were analyzed within an hour after collection. Atmospheric air in the room for breath collection and in the laboratory was also collected and profiled to identify the ambient VOCs which were further used to normalize the VOC values by subtracting the ambient VOCs from the measured VOCs in the breath of participants.

### VOC measurements

**Figure 2:**
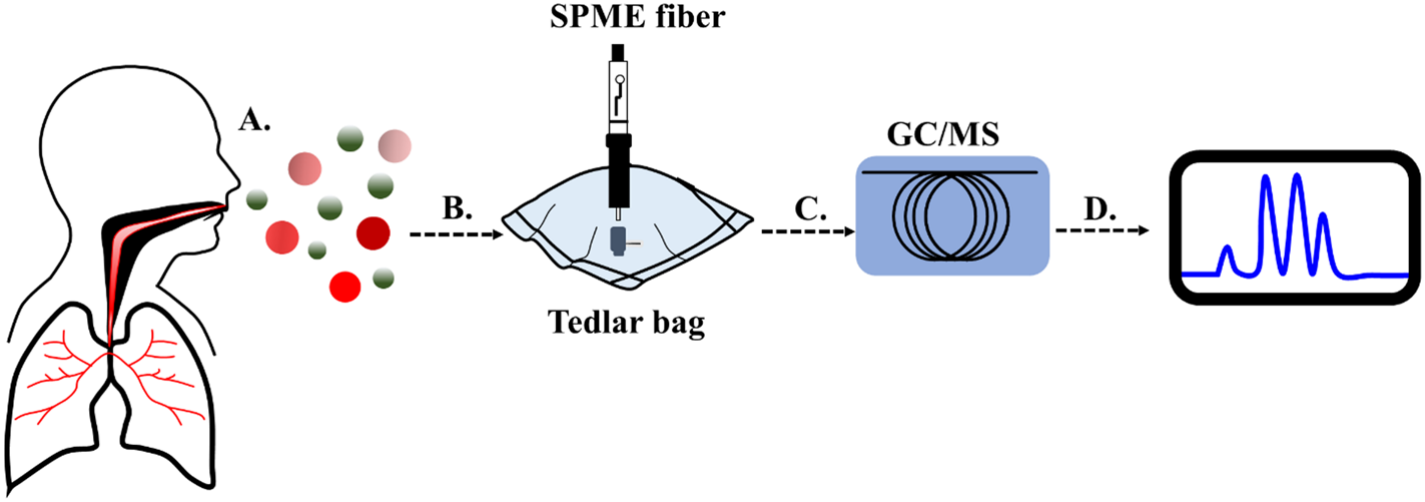
Breath sample was collected using a Tedlar Bag (**A**); Sample was extracted with solid-phase microextraction (SPME) technique (**B**); The compounds were identified using Gas chromatography-Mass spectrometry (GC–MS) (**C**); and Chromatogram was generated (**D**).

The VOCs were profiled by an untargeted metabolomics approach on gas chromatography-mass spectrometry (GC–MS) (Agilent 7000D GC–MS, Triple Quadrupoles system (7890B GC/5975 MS system), Agilent Technologies, Santa Clara, CA, USA), equipped with a CP-Porabond-Q (25 m × 0.25 m × 3 µm) PLOT column (Agilent Technologies, CA, USA)^23^. The schematic diagram of the study is shown in Fig. 2. Breath samples were extracted from the Tedlar bag using Solid Phase Microextraction (SPME) fiber (Carboxen/PDMS fiber 75 µm, needle size 24G, Agilent Technologies, CA, USA) which was conditioned according to the manufacturer’s instructions before the first use and heated at 250 °C for 10 min before each use. For the extraction, the fiber was introduced into the Tedlar bag through the septum and exposed to breath for 15 min at 25 °C, then removed from the bag and immediately inserted into the injector port of GC–MS for desorption for 2 min. Helium (Ultrahigh Purity grade, Lab solution and Engineering Co. Ltd., Nonthaburi, Thailand) was used as a carrier gas at a flow rate of 1 ml/min. Tuning and calibration were performed to ensure that the mass spectrometer was working properly.

The GC–MS analysis was performed using the modified method of Ligor et al*.*^24^. The splitless mode was used with an inlet temperature of 200 °C. The GC oven temperature program was set at 40 °C for 2 min at initial step, ramped to 140 °C at 10 °C/min, followed by an increase to 270 °C at 5 °C/min and held for 5 min. The temperature of the ion source and transfer line was 230 °C and 280 °C, respectively. The electron ionization (EI) was 70 eV. The full scan mode was carried out on MS analysis. The mass range was scanned from m/z 30–300.

For data pre-processing step, Agilent MassHunter software was used for spectral deconvolution and area under the curve (AUC) calculation. Peak picking and identification of VOCs were done by comparison of both mass spectra and retention index (RI) with compounds in the National Institute of Standards and Technology (The NIST 14 mass spectrometry database, Gaithersburg, USA). The criteria acceptance for compound identification was matching score of ≥ 80% (high spectral similarity) and RI value difference of ≤ 20 units between the calculated RI and the database values (Supplemental method).

Because this study used exhaled breath samples that were required to perform GC–MS analysis within the same day of sample collection, we were able to recruit only 3–5 participants per day. For this reason, we could not run all the 242 samples at one time. AUC of the identified compounds were subtracted by blank collected from the room for sample collection each time. Calculated AUC data of each subject were put into the excel file day-by-day. After collecting all the data, alignment was done by comparing AUC of each VOC identified from the NIST database.

### Data analysis

Baseline characteristics of cases and controls were compared using the independent t-test and Pearson’s Chi-square test for continuous and categorical variables, respectively. Before analysis, the concentration values of metabolites were normalized by log_2_ transformation. Levels of VOCs between the 2 groups were compared using t-test.

A Support Vector Machine (SVM) algorithm was then applied to determine the number of VOCs in a combination that provided the greatest accuracy^25^. SVM algorithm created a classification boundary between cases and control groups using the VOCs features. Radial basis function (RBF) kernel was used to create a non-linear classifier. The RBF kernel reduces the chance of overfitting by dimensional reduction. Moreover, we also searched all possible combinations of VOCs to reduce the number of features in the combination to avoid overfitting.

Given the imbalanced numbers of cirrhotic patients and healthy volunteers in the control group, we applied a Synthetic Minority Oversampling Technique (SMOTE) for synthesizing samples in the minority class, which reduced the impact of an imbalanced number of the 2 groups of controls that would cause an inappropriate classification. Indeed, SMOTE creates synthetic samples from existing examples and their nearest neighbors. Thus, the new synthetic samples can overlap with majority class. We therefore used RBF kernel function. The RBF kernel implicitly maps data from input feature space into the Reproducing Kernel Hilbert Space (RKHS) whose dimension is usually much higher than that of the input space. The combination of high-dimensionality and non-linear mapping make that the data that are close to each other in input space may be very far from each other in RKHS. The SVM algorithm explores data in this RKHS in order to create proper class boundary even when classes are tightly overlapped in the input space.

The combination of VOCs that had good performance in isolating cases from controls were further identified. In this analysis, we included only the VOCs that were found in > 5% of the total samples (64 out of the 89 VOCs). The entire patient cohort was divided into 2 independent sets (training n = 152, test n = 56). The training set (61 HCC, 68 cirrhosis, and 23 healthy controls) was used to generate the combinations of VOCs. A leave-one-out cross-validation was performed in the training set. The combinations with the best accuracy, sensitivity and specificity were selected and evaluated for their performance using the test set (36 HCC, 10 cirrhosis and 10 healthy controls).

Next, an association between VOCs profile and HCC stages was determined. The SVM algorithm formed a hyperplane which acted as a boundary between HCC and controls. We hypothesized that if a data point representing an HCC patient is farther away from the boundary, the patient might have a more advanced stage of HCC. To test this hypothesis, we subgrouped HCC patients in the training set by BCLC stages, and then calculated a mean distance of data points in each BCLC stage to the boundary.

We further investigated whether VOCs can be used for detection of early stage HCC. In this analysis, 43 patients with early HCC (BCLC stages 0 and A) and 111 controls were included. Sensitivity, specificity, accuracy and a receiver operating characteristic (ROC) curve of VOCs were estimated. The performance of serum AFP at the cutoff of ≥ 20 ng/mL for detecting early HCC was also evaluated. Sensitivity and specificity of the VOCs and AFP were compared using McNemar test. A *p* value of < 0.05 was considered statistically significant.

Lastly, pre- and post-treatment VOCs levels of 34 HCC patients were compared using paired t test. Changes in VOC levels between treatment response and non-response groups were compared using Mann–Whitney U test.

## Results

### Baseline characteristics

Table 1 displays baseline characteristics of study groups. The number of HCC patients with BCLC stage 0, A, B, C and D were 12 (12.4%), 31 (32.0%), 23 (23.7%), 23 (23.7%) and 8 (8.3%), respectively. Age, gender, etiology of chronic liver diseases were not statistically different between cases and controls (*p* > 0.05). Proportion of individuals with Child–Pugh class A, B and C cirrhosis were significantly different between the 2 groups. The HCC group had significantly higher levels of total bilirubin, aspartate aminotransferase, alkaline phosphatase, and AFP, but lower albumin levels than the control group. In the HCC group, only proportions of patients with underlying non-alcoholic fatty liver diseases were significantly different among patients with stages 0-D HCC, while other factors, including age, gender, smoking and alcohol status, proportions of patients with chronic viral hepatitis B/C infection and diabetes were not statistically different among patients with different stages (Supplemental table 1).

**Table 1:** Baseline characteristics and clinical data.

Variables	Cases (n = 97)	Controls (n = 111)	*P*
Age (mean ± sd.)	61.2 ± 11.6	60.2 ± 10.7	0.52
Male, N (%)	72 (74.2%)	88 (79.3%)	0.39
Smoking, N (%)	27 (27.8%)	27 (24.3%)	0.57
Alcohol consumption, N (%)	41 (42.3%)	36 (32.4%)	0.14
Cirrhosis, N (%)	94 (96.9%)	78 (70.3%)	
Child–Pugh class, N (%)			0.001
A	66/94 (70.2%)	72/78 (92.3%)	
B	18/94 (19.1%)	6/78 (7.7%)	
C	10/94 (10.6%)	0/78 (0.0%)	
Chronic viral hepatitis B infection, N (%)	33 (34.0%)	28 (25.2%)	0.57
Chronic viral hepatitis C infection, N (%)	33 (34.0%)	37 (33.3%)	0.92
Non-alcoholic fatty liver disease (NAFLD), N (%)	13 (13.4%)	27(24.3%)	0.050
Diabetes mellitus, N (%)	23 (23.7%)	36 (32.4%)	0.23
Albumin (g/dL), mean ± SD	3.6 ± 0.8	3.6 ± 1.3	0.81
Total bilirubin (mg/dL), mean ± SD	1.6 ± 1.8	0.9 ± 0.6	< 0.001
Aspartate aminotransferase (U/L), mean ± SD	94.4 ± 110.6	45.8 ± 45.6	< 0.001
Alanine aminotransferase (U/L), mean ± SD	54.4 ± 52.7	42.2 ± 41.1	0.06
Alkaline phosphatase (U/L), mean ± SD	148.9 ± 138.5	96.8 ± 71.3	0.001
Alpha fetoprotein (ng/mL), median (IQR)	44.15 (1,514)	3.09 (4)	0.037

### Analysis of exhaled volatile organic compounds between cases and controls

According to the Metabolomics Standards Initiative guidelines^26^, most of the VOCs identified in this study were MSI level 2 (putative annotated compounds). Of the 64 VOCs included in the analysis (Supplemental Table 2), 18 had significantly different levels between cases and controls (*p* < 0.05) (Supplemental Table 3).

#### Optimal combination of VOCs for classification

**Figure 3:**
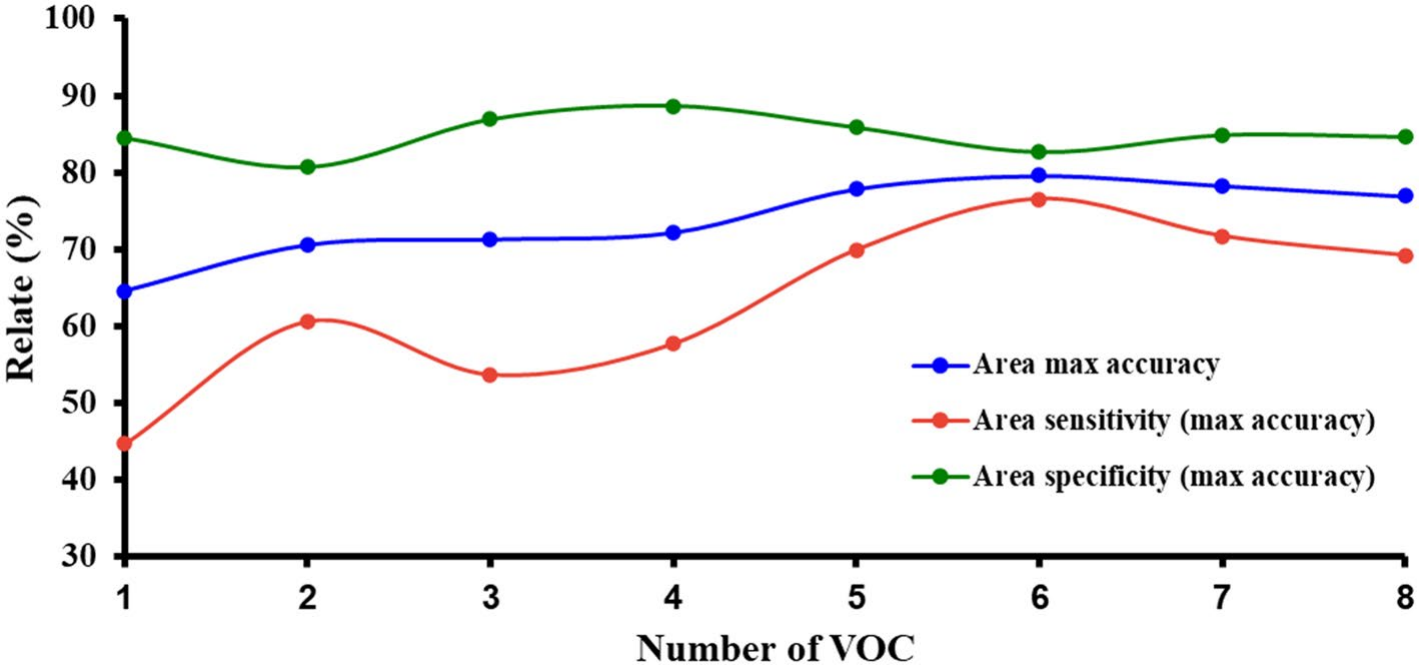
Performance of the number of VOCs in combinations for HCC diagnosis.

We determined the optimal number of VOCs that provided the best performance of the model for differentiating between cases and controls. We found that the accuracy, sensitivity and specificity of the model improved with an increased number of VOCs included in the model. The accuracy and sensitivity reached its maximum value with 6 VOCs in the model, while specificity reached its highest level (87.6%) with 4 VOCs in the model. Since the specificity 6 VOCs model (82.7%) was relatively similar to the 4 VOCs combination, we selected the 6 VOCs model for all accuracy, sensitivity, and specificity combinations for classifying cases and controls (Fig. 3).

#### Performance of VOC combination for HCC diagnosis

To determine the best combination of 6 VOCs for diagnosis of HCC, the accuracy, sensitivity and specificity of each combination were estimated. The combination of 6 VOCs including acetone, 1,4-pentadiene, methylene chloride, benzene, phenol and allyl methyl sulfide provided the highest accuracy of 79.6%, with a sensitivity and specificity of 76.5% and 82.7%, respectively in training set (Table 2). We also determined the combinations of VOCs that provided the highest sensitivity and highest specificity. The combination including acetic acid, methyl ester, methylene chloride, phenol, benzene, cyclopentane and pentane provided the highest sensitivity of 98% (Table 2), while the model including camphene, cyclopentane, methyl, 2-pentanone, dimethyl sulfide, acetonitrile and cyclopentane,1,3-dimethyl provided the highest specificity of 100% (Table 2).

**Table 2:** Top 10 accuracy, sensitivity and specificity -based combinations of VOCs.

Rank of accuracy	1	2	3	4	5	6	7	8	9	10
Accuracy	**0.796**	**0.786**	**0.781**	**0.781**	**0.781**	**0.781**	**0.781**	**0.776**	**0.776**	**0.776**
Sensitivity	0.765	0.714	0.694	0.724	0.724	0.714	0.714	0.714	0.653	0.653
Specificity	0.827	0.857	0.867	0.837	0.837	0.847	0.847	0.837	0.898	0.898
VOCs	Acetone	Acetone	Acetone	Acetone	Acetone	Acetone	Acetone	Acetone	Acetone	Acetone
	1,4-Pentadiene	1,4-Pentadiene	1,4-Pentadiene	1,4-Pentadiene	1,4-Pentadiene	1,4-Pentadiene	1,4-Pentadiene	1,4-Pentadiene	n-Hexane	n-Hexane
	Phenol	Phenol	Phenol	Phenol	Phenol	Phenol	Phenol	Phenol	Dimethyl sulfide	Dimethyl sulfide
	Methylene chloride	Methylene chloride	Methylene chloride	Methylene chloride	Methylene chloride	Methylene chloride	Methylene chloride	Methylene chloride	1-Propene	1-Propene
	Allyl methyl sulfide	Allyl methyl sulfide	Allyl methyl sulfide	Allyl methyl sulfide	Allyl methyl sulfide	Allyl methyl sulfide	Allyl methyl sulfide	Allyl methyl sulfide	N,N-Dimethylacetamide	N,N-Dimethylacetamide
	Benzene	Camphene	D-Limonene	Cyclopentane, methyl	Pentane	Cyclopentane	Cyclopentane,1,3-dimethyl	Camphor	Camphor	Camphor

Rank of sensitivity	1	2	3	4	5	6	7	8	9	10
Accuracy	0.520	0.520	0.520	0.520	0.520	0.520	0.520	0.520	0.515	0.515
Sensitivity	**0.980**	**0.980**	**0.980**	**0.980**	**0.980**	**0.980**	**0.980**	**0.980**	**0.980**	**0.980**
Specificity	0.061	0.061	0.061	0.061	0.061	0.061	0.061	0.061	0.051	0.051
VOCs	Acetic acid, methyl ester	Acetic acid, methyl ester	Acetic acid, methyl ester	Acetic acid, methyl ester	Acetic acid, methyl ester	Acetic acid, methyl ester	Acetic acid, methyl ester	Acetic acid, methyl ester	Acetic acid, methyl ester	Acetic acid, methyl ester
	Methylene chloride	Methylene chloride	Methylene chloride	Methylene chloride	Methylene chloride	Methylene chloride	Methylene chloride	Methylene chloride	Methylene chloride	Methylene chloride
	Phenol	Dimethyl sulfide	Dimethyl sulfide	Dimethyl sulfide	Dimethyl sulfide	Dimethyl sulfide	Dimethyl sulfide	2-Pentanone	Dimethyl sulfide	Dimethyl sulfide
	Benzene	1-Propene	1-Propene	1-Propene	1-Propene	1-Propene	1-Propene	Camphor	1-Propene	1-Propene
	Cyclopentane	Benzene	Cyclopentane	2-Pentanone	Cyclopentane	Pentane	Cyclopentane	Cyclopentane	Pentane, 2-methyl-	Cyclopentane
	Pentane	Pentane	Pentane, 2-methyl-	Pentane, 2-methyl-	Pentane	2-Pentanone	2-Pentanone	Cyclopentane,1,3-dimethyl-	2-Butanone	2-Butanone

Rank of specificity	1	2	3	4	5	6	7	8	9	10
Accuracy	0.566	0.556	0.551	0.546	0.546	0.546	0.536	0.536	0.526	0.526
Sensitivity	0.133	0.112	0.102	0.092	0.092	0.092	0.071	0.071	0.051	0.051
Specificity	**1.000**	**1.000**	**1.000**	**1.000**	**1.000**	**1.000**	**1.000**	**1.000**	**1.000**	**1.000**
VOCs	Camphene	Camphene	Camphene	Camphene	Camphene	Camphene	Camphene	Camphene	Camphene	Camphene
	Cyclopentane, methyl	Cyclopentane, methyl	Cyclopentane, methyl	Cyclopentane, methyl	Cyclopentane, methyl	Cyclopentane, methyl	Cyclopentane, methyl	Methylene chloride	Benzene	Acetonitrile
	2-Pentanone	2-Butanone	Acetonitrile	Benzene	2-Pentanone	2-Pentanone	2-Pentanone	2-Pentanone	2-Pentanone	2-Pentanone
	Dimethyl sulfide	Dimethyl sulfide	Dimethyl sulfide	Methylene chloride	Methylene chloride	Cyclopentane	Dimethyl sulfide	Dimethyl sulfide	Pentane	Pentane
	Acetonitrile	Phenol	Phenol	Cyclopentane	Benzene	Acetic acid, methyl ester	Phenol	Cyclopentane	Phenol	Phenol
	Cyclopentane,1,3-dimethyl	Cyclopentane,1,3-dimethyl	n-Hexane	Cyclopentane,1,3-dimethyl	Cyclopentane,1,3-dimethyl	Cyclopentane,1,3-dimethyl	n-Hexane	n-Hexane	Acetonitrile	Cyclopentane

Further, we extracted the VOCs that were frequently present in the top 10 of VOCs combinations (Table 3). We observed that acetone, methylene chloride, phenol, 1,4-pentadiene and allyl methyl sulfide were commonly used in accuracy-based combinations. When the best accuracy-based model was tested in the test set, the model provided an accuracy of 55.4%, with a sensitivity and specificity of 44.0% and 75.0%, respectively.

**Table 3:** The frequency of VOCs commonly identified in the top 10 accuracy-, sensitivity- and specificity-based combinations.

Accuracy			Sensitivity			Specificity		
Rank	VOC	Count	Rank	VOC	Count	Rank	VOC	Count
1	Acetone	10	1	Acetic acid, methyl ester	10	1	Camphene	10
2	Methylene chloride	8	2	Methylene chloride	10	2	Cyclopentane, methyl-	7
3	Phenol	8	3	Dimethyl sulfide	8	3	2-Pentanone	7
4	1,4-Pentadiene	8	4	1-Propene	8	4	Dimethyl sulfide	5
5	Allyl methyl sulfide	8	5	Cyclopentane	6	5	Phenol	5

#### Performance of VOCs for diagnosis of early HCC

Among the VOCs studied, d-limonene provided the highest sensitivity of 62.8%. The sensitivity of d-limonene was significantly higher than 25.6% sensitivity of the AFP (*p* = 0.002). However, d-limonene had a significantly lower specificity than AFP (51.8% vs. 74.4%, *p* ≤ 0.001). The accuracies of d-limonene and AFP were 54.9% and 76.0%, with AUCs of 0.613 and 0.605, respectively.

### Association between HCC stages and distances from SVM boundary

**Figure 4:**
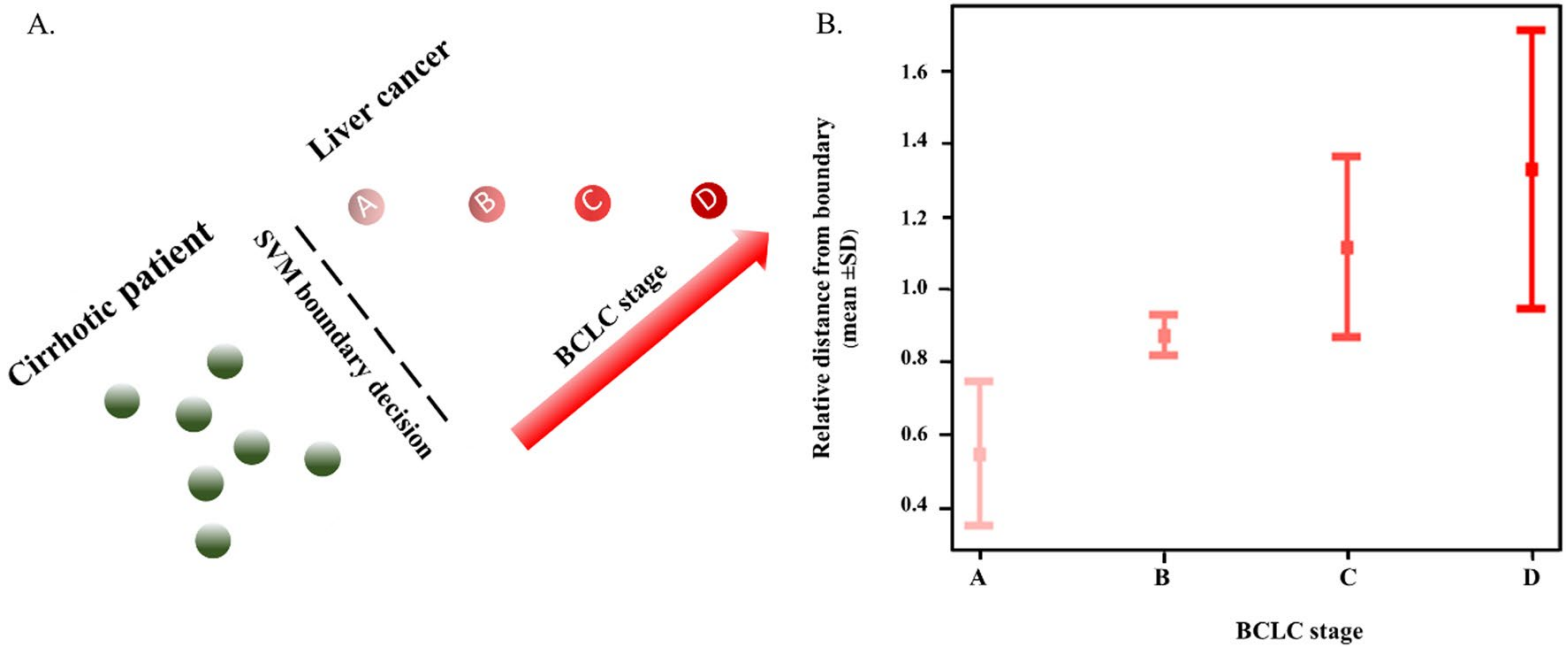
Schematic figure of correlation between HCC stages classified by the Barcelona-Clinic Liver Cancer (BCLC) staging system and distance from the support vector machine (SVM) classification boundary (4A). The relative distance from the SVM boundary of the HCC stages (4B).

We subgrouped HCC patients by BCLC stages and then calculated a mean distance for each data point in each BCLC stage to the boundary. The boundary used in this analysis was formed by the SVM model^27^ that incorporated the combination of 6 VOCs that yielded the highest accuracy, including acetone, 1,4-pentadiene, methylene chloride, benzene, phenol and allyl methyl sulfide. Results showed that the distance from the classification boundary increased as the stage of HCC advanced (Fig. 4). Mean ± SD distances from the boundary to the data points representing HCC patients with BCLC stage A, B, C and D were 0.55 ± 0.20, 0.87 ± 0.06, 1.11 ± 0.25 and 1.33 ± 0.39 units, respectively.

### Changes of VOCs after HCC treatment

Of the 34 HCC patients selected for examination of post-treatment VOCs, 20 and 14 patients underwent transarterial chemoembolization (TACE), and percutaneous local ablative therapy (PLAT) with radiofrequency ablation or microwave ablation respectively. After treatment, the level of acetone significantly decreased from 94.42 ± 58.00 to 40.90 ± 46.61 (× 10^6^ arbitrary unit (AU)), *p* < 0.001, while the levels of dimethyl sulfide and butane significantly increased from 0.62 ± 1.56 to 1.84 ± 2.56 and 0.08 ± 0.48 to 1.63 ± 4.27 (× 10^6^ AU), *p* = 0.003 and 0.045, respectively.

After treatment, 22 (64.7%) patients (11 TACE, 11 PLAT) responded to treatment, while other 12 (35.3%) patients (9 TACE, 3 PLAT) had remaining viable tumors. The magnitude of reduction in acetone levels after treatment in the response group was significantly greater than that of the non-response group, i.e. 73.38 ± 56.76 vs. 17.11 ± 58.86 (× 10^6^ AU), *p* = 0.006. Using a cutoff of decreased level of acetone at 35.9 × 10^6^ AU, it provided a sensitivity, specificity and accuracy of 77.3%, 83.3% and 79.4%, with an AUC of 0.784, for differentiating between responders and non-responders.

Among 20 patients treated with TACE, there were 11 (55%) responder and 9 (45%) non-responders. The increased level of dimethyl sulfide was significantly greater in the response group, i.e. 2.2 ± 2.6 vs. 0.3 ± 0.9 (x 10^6^ AU), *p* = 0.046. Responders had a decreased level of acetone greater than non-responders but the difference did not reach statistical significance (61.1 ± 38.8 vs. 31.1 ± 53.1 AU, *p* = 0.175). Of the 14 patients who were treated with PLAT, 11 (79%) responded to the treatment while 3 (21%) did not respond. Those who responded to PLAT had decreased levels of acetone and allyl methyl sulfide while the non-responders had increased level of both VOCs after therapy. The altered levels of acetone and allyl methyl sulfide in the response and non-response group were significantly different i.e. 85.7 ± 70.2 vs. − 25.0 ± 65.0 (× 10^6^ AU) and 3.7 ± 7.4 vs. − 6.7 ± 4.1 (× 10^6^ AU), for acetone and allyl methyl sulfide, *p* = 0.011 and 0.038, respectively.

## Discussion

In this study, we determined optimal combinations of VOCs for HCC diagnosis based on the highest accuracy, sensitivity and specificity using SVM classification. The accuracy-based combination is useful for diagnosis, while the sensitivity-based combination is useful to identify patients with high risk for HCC development. The specificity-based combination is useful for identifying individuals who are unlikely to have HCC in order to avoid further unnecessary investigation. The accuracy-based combination was correlated with the HCC stage. We identified a VOC for diagnosis of early HCC and the VOCs that had a better sensitivity than serum tumor marker AFP for diagnosis of early HCC. After treatment with TACE and PLAT, the levels of VOCs were significantly altered, and the decreased level of acetone predicted response to therapy with satisfactory performance. These findings suggest that VOCs had potential to be biomarkers for HCC diagnosis and for monitoring therapeutic response.

The VOCs identified in this study were consistent with those previously reported in other cancers^17,23,28–32^ VOCs are products of cellular metabolic activity. The energy metabolism of cancer cells differs from that of normal cells. The malignant cells have a propensity to produce adenosine triphosphate (ATP) via glycolysis rather than oxidative phosphorylation, so called aerobic glycolysis. The metabolic interactions between cancer cells and other components in microenvironment, particularly cancer-associate fibroblasts (CAFs), are also important for cancer cell proliferation and survival. Aerobic glycolysis is enhanced in CAFs, resulting in the production of lactate, ketone bodies and free fatty acids^33^. These metabolites serve as nutrients for cancer cells and promote tumor growth and metastasis. Some VOCs identified in the current study are known to be metabolites from these aberrant metabolisms, for example, acetone—a main type of ketone bodies, and 1,4-pentadiene—a product of free fatty acid. Acetone was identified as one of the most important features for classifying HCC cases from controls as well as for monitoring response to therapy. The level of acetone increased when HCC was developed and significantly decreased after the treatment.

One of the strength of our study is the use of a SVM algorithm to identify the best combination of VOCs for various clinical purposes. Since cancer cells are heterogeneous in each tumor and among patients, it is unlikely that a single biomarker can be a perfect biomarker for early detection, diagnosis and monitoring treatment response. The VOCs combination with the highest accuracy was correlated with the stage of disease. We found that the accuracy of VOCs in the test set was worse than that of the training set. The lower accuracy was likely driven by the differences in baseline characteristics between the 2 cohorts, particularly the stages of HCC. The test set had significantly more proportion of patients with early HCC (BCLC stages 0 and A) than the training set, i.e. 23/36 (63.9%) vs. 20/61 (32.8%), *p* = 0.021, (Supplemental Table 4). To improve the performance of the predictive model for early HCC, a further study with a larger number of patients with early stage HCC is needed. Most of our controls had underlying cirrhosis, one of the main risk factors for HCC, thus our control group was more representative of individuals who would be the target population in clinical practice. Because this study was conducted in a single center, validation of these findings with other independent cohorts is warranted before applying the exhaled VOCs in practice. Although the performance of VOCs observed in our study was not better than ultrasound for HCC detection, its sensitivity was greater than the AFP, the main serum tumor marker used in clinical practice. It is interesting to further investigate the usefulness of VOCs as an adjunctive tool to improve the performance of ultrasound for HCC detection. Some exogenous confounding factors including diet, smoking, and alcohol drinking may potentially affect the VOC profiles. However, we tried to minimize the effect of these confounders by having the participants fasted, and stopped smoking and drinking before breath collection. The numbers of participants who smoked and drank alcohol were not significantly different between cases and controls. Thus, we believe that these confounders minimally impact the findings of the study. The non-invasive nature of breath testing and high acceptance rate among patients does bode well for seamless clinical implementation if future studies continue to show high efficacy.

## Conclusion

Exhaled VOCs profiles in HCC patients are different from individuals without HCC and may potentially be used as biomarkers for HCC diagnosis and treatment.

## Supplementary Information

Supplementary Information.
