# Supplementary material for: Exhaled volatile organic compounds for diagnosis of hepatocellular carcinoma
Source: Sci Rep. 2022 Mar 29;12:5326. doi: 10.1038/s41598-022-08678-z (PMC8964758; doi:10.1038/s41598-022-08678-z)
Supplement: Supplementary file 1 — Supplementary Information. [file 41598_2022_8678_MOESM1_ESM.docx]

**Supplement Material**

**Exhaled volatile organic compounds for diagnosis of hepatocellular carcinoma**

**Supplemental method**

The RI values were calculated from the retention time of n-alkane series (C7-C30) (Supelco, Sigma-Aldrich, PA, USA) using the following equation (1):


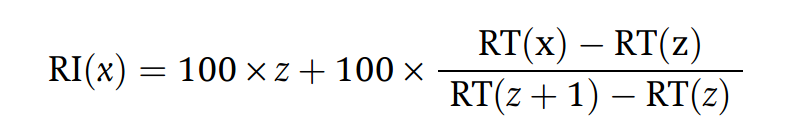


where RI (x) is the retention index of the unknown compound x,

z is the number of carbon atoms of the n-alkane eluted before the unknown compound x,

z + 1 is the number of carbon atoms of the n-alkane eluted after the unknown compound x,

RT (x) is the retention time of the unknown compound x,

RT (z) the retention time of the n-alkane eluted before the unknown compound x,

RT (z + 1) is the retention time of n-alkane eluted after the unknown compound x.

RI values calculated from a PLOT column in this study were ≤20% different from the RIs of ordinary capillary siloxane-based column (1), which can be implied that column transferability was accurate.

**References**

1. Bianchi F, Careri M, Mangia A, Musci M. Retention indices in the analysis of food aroma volatile compounds in temperature-programmed gas chromatography: database creation and evaluation of precision and robustness. J Sep Sci. 2007;30(4):563-72.

**Supplemental Table 1.** Baseline characteristics of HCC patients with different stages

| Variables | HCC stage | | | | | P |
| --- | --- | --- | --- | --- | --- | --- |
|  | 0 | A | B | C | D |  |
| Number of patients | 12 | 31 | 23 | 23 | 8 |  |
| Age (mean±SD) | 68.0±10.6 | 61.3±13.5 | 60.9±12.3 | 58.7±9.0 | 58.4±7.2 | 0.22 |
| Male, n (%) | 8 (66.7%) | 24 (77.4%) | 18 (78.3%) | 16 (69.6%) | 6 (75.0%) | 0.91 |
| Smoking, n (%) | 3 (25.0%) | 7 (22.6%) | 8 (34.8%) | 4 (17.4%) | 5 (62.5%) | 0.13 |
| Alcohol consumption, n (%) | 3 (25.0%) | 11 (35.5%) | 9 (39.1%) | 11 (47.8%) | 7 (87.5%) | 0.06 |
| Chronic viral hepatitis B infection, n (%) | 3 (25.0%) | 12 (38.7%) | 6 (26.1%) | 8 (34.8%) | 4 (50.0%) | 0.68 |
| Chronic viral hepatitis C infection, n (%) | 4 (33.3%) | 12 (38.7%) | 10 (43.5%) | 5 (21.7%) | 2 (25.0%) | 0.55 |
| Non-alcoholic fatty liver disease, n (%) | 4 (33.3%) | 4 (12.9%) | 5 (21.7%) | 0 (0.0%) | 0 (0.0%) | 0.016 |
| Diabetes, N (%) | 4 (33.3%) | 7 (22.6%) | 6 (26.1%) | 5 (21.7%) | 1 (12.5%) | 0.81 |

**Supplemental Table 2.** Listing of 64 VOCs included in the analysis

| **Retention time** (**min.**) | **Compounds** | **Chemical Abstracts Service** | **R Match** |
| --- | --- | --- | --- |
| 9.631 | ethanol | 64-17-5 | 981 |
| 10.411 | bromochlorodifluoromethane | 353-59-3 | 950 |
| 10.445 | acetonitrile | 75-05-8 | 882 |
| 10.471 | methyl isocyanide | 593-75-9 | 877 |
| 10.884 | butane | 106-97-8 | 946 |
| 12.112 | acetone | 67-64-1 | 975 |
| 12.215 | methylene chloride | 75-09-2 | 952 |
| 12.600 | isopropyl alcohol | 67-63-0 | 973 |
| 12.713 | dimethyl sulfide | 75-18-3 | 969 |
| 13.178 | acetic acid, methyl ester | 79-20-9 | 945 |
| 13.194 | acetic acid | 64-19-7 | 967 |
| 13.567 | 1-propanol | 71-23-8 | 924 |
| 13.977 | butane, 2-methyl- | 78-78-4 | 838 |
| 14.134 | 1,4-pentadiene | 591-93-5 | 900 |
| 14.280 | sevoflurane | 100308-79-8 | 882 |
| 14.595 | pentane | 109-66-0 | 936 |
| 15.967 | acetic acid ethenyl ester | 108-05-4 | 912 |
| 15.975 | 2,3-butanedione | 431-03-8 | 935 |
| 16.009 | furan, 3-methyl- | 903-27-8 | 937 |
| 16.103 | 2-butanone | 78-93-3 | 935 |
| 16.743 | ethane, (methylthio)- | 624-89-5 | 896 |
| 16.848 | ethyl Acetate | 141-78-6 | 952 |
| 18.041 | pentane, 2-methyl- | 107-83-5 | 904 |
| 18.347 | pentane, 3-methyl- | 96-14-0 | 955 |
| 18.493 | benzene | 71-43-2 | 973 |
| 18.752 | cyclopentane | 287-92-3 | 809 |
| 18.890 | n-hexane | 110-54-3 | 930 |
| 19.735 | cyclohexane | 110-82-7 | 923 |
| 20.296 | acetoin | 513-86-0 | 936 |
| 20.394 | 2-pentanone | 107-87-9 | 897 |
| 20.399 | furan, 2,5-dimethyl- | 625-86-5 | 946 |
| 20.554 | allyl methyl sulfide | 10152-76-8 | 924 |
| 21.289 | 1-propene | 115-07-1 | 898 |
| 21.330 | propane, 1-(methylthio)- | 3877-15-4 | 856 |
| 21.484 | pentane,2,4-dimethyl- | 108-08-7 | 911 |
| 21.921 | cyclopentane, methyl- | 96-37-7 | 938 |
| 22.258 | cyclopentane, 1,1-dimethyl- | 1638-26-2 | 900 |
| 22.269 | cyclopentane,1,2-dimethyl- | 2452-99-5 | 898 |
| 22.545 | hexane, 2-methyl- | 591-76-4 | 905 |
| 22.657 | hexane, 3-methyl- | 589-34-4 | 928 |
| 23.308 | toluene | 108-88-3 | 959 |
| 23.467 | heptane | 142-82-5 | 872 |
| 23.520 | cyclohexane, methyl- | 108-87-2 | 903 |
| 25.076 | n,n-dimethylacetamide | 127-19-5 | 952 |
| 27.571 | ethylbenzene | 100-41-4 | 923 |
| 27.654 | phenol | 108-95-2 | 946 |
| 27.806 | 4-Methyl-2,4-bisp-hydroxyphenylpent-1-ene (2TMS derivative) | 13464-24-9 | 925 |
| 32.278 | benzene, 1,3-dimethyl- | 108-38-3 | 860 |
| 32.659 | d-α-pinene | 80-56-8 | 915 |
| 33.023 | 3,4-dimethoxycinnamic acid |  |  |
| 33.636 | camphor | 76-22-2 | 850 |
| 33.728 | cyclohexene, 4-methylene-1-(1-methylethyl)- | 99-84-3 | 866 |
| 33.730 | sabinen | 3387-41-5 | 861 |
| 34.183 | β-pinene | 127-91-3 | 872 |
| 35.100 | 3-carene | 13466-78-9 | 929 |
| 35.938 | d-limonene | 5989-27-5 | 947 |
| 39.002 | eucalyptol | 470-82-6 | 903 |
| 39.748 | (+)-2-bornanone | 464-49-3 | 899 |
| 40.138 | l-borneol | 507-70-0 | 853 |
| 40.212 | l-menthone | 14073-97-3 | 862 |
| 40.224 | p-menthone | 89-80-5 | 842 |
| 40.898 | menthol | 2216-51-5 | 838 |
| 40.970 | cyclohexanol, 5-methyl-2-1-(methylethyl)- | 1490-04-6 | 925 |
| 43.001 | styrene | 100-42-5 | 872 |

**Supplemental Table 3.** List of differentially expressed exhaled volatile organic compounds between patients with hepatocellular carcinoma and controls

| **Number** | **VOCs** | ***t*_stat_** | ***p*-value** | **-log10(p)** | **FDR** |
| --- | --- | --- | --- | --- | --- |
| 1 | 1,4-pentadiene | -5.7548 | 3.449 x10^-8^ | 7.4622 | 2.7598 x10^-6^ |
| 2 | Benzene | 5.5077 | 1.1761 x10^-7^ | 6.9296 | 4.7043 x10^-6^ |
| 3 | Acetone | -5.4041 | 1.9466 x10^-7^ | 6.7107 | 5.191 x10^-6^ |
| 4 | Phenol | -4.8525 | 2.5436 x10^-6^ | 5.5945 | 5.0873 x10^-5^ |
| 5 | Allyl methyl sulfide | -4.1016 | 6.0919 x10^-5^ | 4.2152 | 0.00097471 |
| 6 | D-limonene | -3.8435 | 0.00016559 | 3.7810 | 0.0022078 |
| 7 | Dimethyl sulfide | -3.6421 | 0.00034909 | 3.4571 | 0.0039896 |
| 8 | 3,4-Dimethoxycinnamic acid | 3.3646 | 0.00092818 | 3.0324 | 0.0092818 |
| 9 | Methylene chloride | 3.329 | 0.0010477 | 2.9798 | 0.0093128 |
| 10 | Methyl methacrylate | 2.5492 | 0.011591 | 1.9359 | 0.092726 |
| 11 | Camphene | 2.4626 | 0.01469 | 1.8330 | 0.10683 |
| 12 | Cyclopentane, methyl- | -2.3117 | 0.021874 | 1.6601 | 0.14583 |
| 13 | Ethylbenzene | 2.2536 | 0.025372 | 1.5956 | 0.15005 |
| 14 | 1-Propene | -2.2157 | 0.027903 | 1.5544 | 0.15005 |
| 15 | 4-Methyl-2,4-bisp-hydroxyphenylpent-1-ene  (2TMS derivative) | 2.2124 | 0.028135 | 1.5508 | 0.15005 |
| 16 | Sevoflurane | -2.1079 | 0.036358 | 1.4394 | 0.18179 |
| 17 | Hexane, 3-methyl | -2.077 | 0.039155 | 1.4072 | 0.18426 |
| 18 | Furan, 2-methoxy | 2.0401 | 0.042734 | 1.3692 | 0.18993 |

**Supplemental Table 4.** Baseline characteristics of HCC patients in training and test set

| Variables | training set  (n=61) | test set  (n=36) | *P* |
| --- | --- | --- | --- |
| Age (mean ± SD) | 61.3 ± 11.0 | 60.3 ± 12.6 | 0.69 |
| Male, n (%) | 49 (80.3%) | 24 (66.7%) | 0.13 |
| Smoking, n (%) | 13 (21.3%) | 11 (30.6%) | 0.31 |
| Alcohol consumption, n (%) | 25 (41.0%) | 12 (33.3%) | 0.45 |
| Child-Pugh class, n (%)^*^ |  |  | 0.14 |
| A | 42/61 (68.9%) | 24/36 (66.7%) |  |
| B | 12/61 (19.7%) | 6/36 (16.7%) |  |
| C | 7/61 (11.5%) | 3/36 (8.3%) |  |
| BCLC stage, n (%) |  |  | 0.044 |
| 0 | 6/61 (9.8%) | 6/36 (16.7%) |  |
| A | 14/61 (23.0%) | 17/36 (47.2%) |  |
| B | 19/61 (31.2%) | 4/36 (11.1%) |  |
| C | 16/61 (26.2%) | 7/36 (19.4%) |  |
| D | 6/61 (9.8%) | 2/36 (5.6%) |  |
| Chronic viral hepatitis B infection, n (%) | 18 (29.5%) | 15 (41.7%) | 0.22 |
| Chronic viral hepatitis C infection, n (%) | 23 (37.7%) | 10 (27.8%) | 0.32 |
| Non-alcoholic fatty liver disease (NAFLD), n (%) | 7 (11.5%) | 5 (13.9%) | 0.53 |
| Diabetes mellitus, n (%) | 16 (26.2%) | 7 (19.4%) | 0.54 |
| Albumin (g/dL), mean ± SD | 3.5 ± 0.6 | 3.7 ± 1.0 | 0.30 |
| Total bilirubin (mg/dL), mean ± SD | 1.7 ± 1.7 | 1.6 ± 2.1 | 0.68 |
| Aspartate aminotransferase (U/L), mean ± SD | 105.3 ± 112.9 | 75.2 ± 106.0 | 0.20 |
| Alanine aminotransferase (U/L), mean ± SD | 60.7 ± 60.3 | 42.9 ± 34.6 | 0.11 |
| Alkaline phosphatase (U/L), mean ± SD | 142.1 ± 74.0 | 159.6 ± 207.8 | 0.55 |
| Alpha fetoprotein (ng/mL), median (IQR) | 17,369.7 ± 81,520.7 | 10,856.0 ± 37,694.0 | 0.67 |

^*^ 3 *patients in the test set did not have cirrhosis*
